# Prevalence & Factors Associated With Acute Kidney Injury in Patients Undergoing Percutaneous Coronary Intervention at a Tertiary Healthcare Facility in Tanzania

**DOI:** 10.7759/cureus.36219

**Published:** 2023-03-16

**Authors:** Faisal Hooda, Nadeem Kassam, Samina Somji, Mandela Makakala, Mariam Noorani, Fatma Bakshi, Robert Mvungi

**Affiliations:** 1 Internal Medicine, Aga Khan University Medical College East Africa, Dar es Salaam, TZA; 2 Pediatric Medicine, Aga Khan University Medical College East Africa, Dar es Salaam, TZA; 3 Interventional Cardiology, Aga Khan Hospital, Dar es Salaam, TZA

**Keywords:** acute kidney injury, sub saharan africa, primary percutaneous coronary intervention (pci), coronary artery disease, contrast-induced acute kidney injury

## Abstract

Background: Coronary artery disease (CAD) is the leading cause of mortality and morbidity globally. Percutaneous coronary intervention (PCI) is a minimally-invasive lifesaving intervention for these patients; however, acute kidney injury (AKI) is a serious complication of the procedure commonly occurring due to radiocontrast-induced nephropathy.

Methods: A retrospective cross-sectional analytical study was carried out at the Aga Khan Hospital, Dar es Salaam (AKH,D), Tanzania. A total of 227 adults who underwent a percutaneous coronary intervention from August 2014 to December 2020 were enrolled. The AKI was defined based on an increase in absolute and rise in percentage creatinine using the Acute Kidney Injury Network (AKIN), and contrast-induced acute kidney injury (CI-AKI) by the Kidney Disease: Improving Global Outcomes (KDIGO) criteria. Bivariable and multivariable logistic regression was utilized to analyze factors associated with AKI and the outcomes of these patients.

Results: Twenty-two of the 227 (9.7%) participants sustained AKI. The majority of the study population was male and of Asian ethnicity. No statistically significant factors were associated with AKI. The in-hospital mortality rate was 9% for the AKI versus 2% for non-AKI groups. The AKI group had a longer hospital stay and required ICU care and organ support including hemodialysis.

Conclusions: Nearly 1-in-10 patients undergoing PCI are likely to develop AKI. The in-hospital mortality rate is x4.5 times higher for patients with AKI post-PCI compared to those without AKI. Further larger studies are recommended to determine factors associated with AKI in this population.

## Introduction

Coronary artery disease (CAD) is the leading cause of mortality and loss of disability-adjusted life years (DALYs) globally. A large fraction of this burden falls on low- and middle-income countries accounting for over seven million deaths annually and is expected to rise exponentially if no strategic measures are taken. Coronary revascularization with percutaneous coronary intervention (PCI) and coronary artery bypass grafting (CABG) is most often offered to patients suffering from CAD not amenable to medical therapy. Despite PCI being considered a minimally invasive lifesaving intervention, acute kidney injury (AKI) post-PCI is a serious but potentially preventable complication.

Acute kidney injury post-PCI is associated with high in-hospital and long-term mortality. Several studies have reported varying incidences of AKI post-PCI ranging between 2% to 30% [1-5]. It is recognized that AKI post-PCI primarily occurs due to radio contrast-induced acute kidney injury (CI-AKI). The risk of AKI increases with the use of higher volumes, hyperosmolar, and higher viscosity of contrast media that are more damaging due to sludging in the renal tubules [1,3]. Alternative mechanisms also exist which include: renal hypoperfusion from cardiac dysfunction, and athero-embolization and PCI-related hemodynamic instability [1,6,7]. Additionally, patients suffering from CAD may have antecedent conditions that make them more vulnerable to AKI, including pre-existing kidney disease, hypertension, diabetes mellitus, heart failure, and anemia [2]. Data from studies done in numerous western countries have yielded varying incidences and different factors associated with AKI post-PCI. Despite the rapid increase in cardiovascular-related morbidity and mortality in Sub-Saharan Africa, there has been a paucity of epidemiological data within the region. Results from a small observational study conducted on a Sudanese cohort reported the use of angiotensin-converting enzyme (ACE) inhibitors to be primarily associated with post-PCI AKI [5].

Existing studies are inconsistent on the prevalence and show conflicting data on factors associated with AKI post-PCI. Over the past decade with great efforts by the public and private sectors in Tanzania, there are now three PCI-capable facilities to date in the country to tackle the rising burden of CAD in the region. With these recent advancements and investments made by the country and PCI being readily available, there is a great need to determine the prevalence of AKI post-PCI in the Sub-Saharan African setting, and to identify factors associated with AKI post-PCI.

## Materials and methods

A retrospective cross-sectional analytical study was conducted at the Aga Khan Hospital, Dar es Salaam (AKH,D), Tanzania-one of the largest private hospitals and the only Joint Commission International (JCI) accredited hospital in the region. The hospital set up its cardiac catheterization laboratory (cath lab) in August 2014. It currently has a 24-hour fully-functional cath lab and a four-bed cardiac critical unit (CCU) equipped with technology to monitor invasive and non-invasive hemodynamics. The hospital also offers standard nephrology care with an eight-bed dialysis unit available within the hospital.

The study approved by the Aga Khan University, East Africa Ethics Review Committee (approval no. AKU/2021/028/fb/06), aimed primarily to determine the prevalence of AKI in patients undergoing PCI at the AKH,D. Secondary outcomes included determining the severity of AKI in patients post-PCI, factors associated with AKI post-PCI, and outcomes of AKI post-PCI.

The study recruited individuals aged ≥18 years and diagnosed with acute and chronic coronary syndromes who underwent PCI from August 2014 through December 2020. Patients with incomplete data and those already on renal replacement therapy were excluded from the study.

The data in this study was collected from the in-hospital US National Cardiovascular Data Registry of Catheterisation- Percutaneous Coronary Intervention (NCDR CathPCI®) database and other hospital records. Data were extracted by experienced junior doctors with working experience in the cardiac unit and verified by the primary investigator for accuracy and completeness. A minimum sample size of 137 was deemed sufficient to power the study to 80% with a 95% confidence interval as shown below:

(N= (( (Z 1-á)⁄2 )^2×P (1-P))/d^2\) where: (Z 1-á)⁄2 )^2=standard normal variate (1.96); P=expected proportion in the population, based on the Sudanese Study (16.9%) [5]; d= relative precision (taken at 5% of P); N= 137 minimum sample size

Patient demographics and clinical data were extracted using patient and medical records and were entered into a spreadsheet on Microsoft Office Excel 2010 (Microsoft Corp., Redmond, WA, USA). Patients were grouped according to the presence of AKI and classified by the contrast-induced acute kidney injury (CI-AKI) by the Kidney Disease: Improving Global Outcomes (KDIGO) and Acute Kidney Injury Network (AKIN) criteria [8-10]. Hospital stay was classified into good and adverse outcomes. A good outcome was considered a stable discharge within three days of PCI. An adverse outcome was indicated by: >3 days of hospital stay, requiring ICU admission, requiring the use of ventilator/vasopressor support, requiring acute hemodialysis, and death prior to discharge.

A descriptive analysis of demographic characteristics was done and presented as percentages while the categorical and continuous outcome variables were analyzed and presented as means and medians. Categorical and continuous variables between the two groups were compared using Fisher’s exact test and the Wilcoxon rank sum test. Any variable with a p-value<0.05 was considered statistically significant. Variables that had a p-value of <0.2 were entered into a multivariable logistic regression model to determine those independently associated with AKI. Odds ratios were calculated to determine the strength of the association and a p-value of <0.05 was considered statistically significant. All analyses were performed using STATA® Software Version 15 (Stata Corp., College Station, TX, USA).

## Results

**Figure 1:**
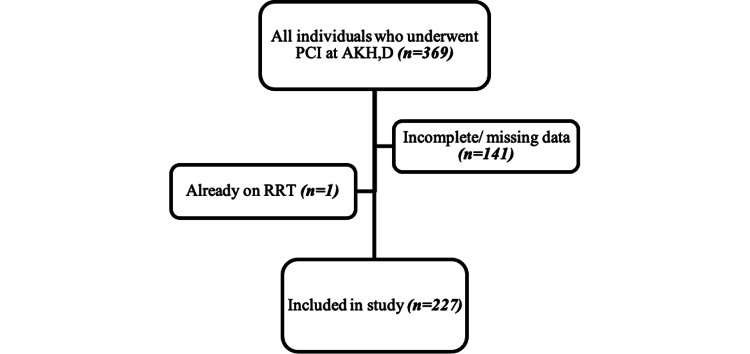
Flowchart of the study PCI: Percutaneous coronary intervention; AKH,D: Aga Khan Hospital, Dar es Salaam; RRT: Renal replacement therapy

A total number of 369 patients underwent PCI at the AKHD from August 2014 to December 2020. Of these participants, 142 were excluded of which 141 had incomplete data and one was an end-stage renal disease (ESRD) patient on hemodialysis prior to admission. A total of 227 participants were enrolled in the study as shown in the flow chart (Figure 1).

The median age amongst our study population was 62 (interquartile range (IQR): 53-70), the majority of whom were male (n=185, 81.5%) and of Asian ethnicity (n= 157, 69.2%). Demographic characteristics are further described in Table 1.

**Table 1:** Demographic characteristics of the study population ^1^Median (IQR) or frequency (%); ^2^Fisher’s exact test, Wilcoxon rank sum test AKI: Acute kidney injury, IQR: Interquartile range

Patient characteristics	Overall n=227^1^	Non-AKI n=205 (90.3%)^1^	AKI n=22 (9.7%)^1^	p-value^2^
Median age (in years)	62 (53,70)	62 (53,70)	64 (56,70)	0.41
Sex				
Male	185 (81.5%)	167 (81.5%)	18 (81.8%)	0.97
Female	42 (18.5%)	38 (18.5%)	4 (18.2%)	
Median BMI, kg/m^2^	28 (25,32)	27 (25,32)	30 (25,34)	0.48
Ethnicity				0.74
Asian	157 (69.2%)	142 (69.3%)	15 (68.2%)	
Black	45 (19.8%)	39 (19.0%)	6 (27.3%)	
Caucasian	17 (7.5%)	16 (7.8%)	1 (4.5%)	
Others	8 (3.5%)	8 (3.9%)	0 (0.0%)	

**Figure 2:**
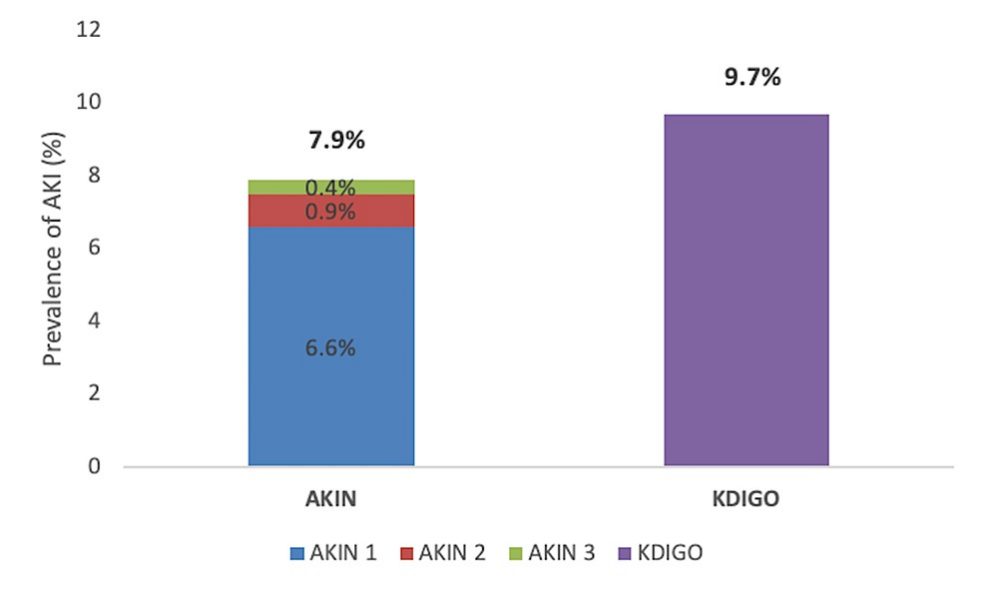
Prevalence of acute kidney injury by severity AKI: Acute kidney injury, AKIN: Acute Kidney Injury Network, KDIGO: Kidney Disease: Improving Global Outcomes

The prevalence of aggregate AKI taking both AKIN and CI-AKI KDIGO criteria was 9.7% (95% confidence interval (CI): 6.2%-14.3%). The prevalence according to the CI-AKI KDIGO criteria alone was 9.7% (95% CI: 6.2%-14.3%) which was higher as compared to the AKIN criteria which found a prevalence of 7.9% (95% CI: 4.8%-12.2%). This was further staged 1-3 as illustrated in Figure 2 below.

Table 2 below highlights the comorbid conditions amongst our study population. The majority of our study population had underlying hypertension (n=165, 72.7%), and diabetes mellitus (n=116, 51.1%). There was no significant difference in comorbid conditions between the group that sustained AKI and those that did not.

**Table 2:** Comorbid conditions in our study population ^1^Median (IQR) or frequency (%); ^2^Fisher’s exact test, Wilcoxon rank sum test AKI: Acute kidney injury, IQR: Interquartile range, ACS: Acute coronary syndrome, CKD: Chronic kidney disease, DM: Diabetes mellitus, HTN: Hypertension, PCI: Percutaneous coronary intervention

Comorbid conditions	Overall n=227^1^	Non-AKI n=205 (90.3%)^1^	AKI n=22 (9.7%)^1^	p-value^2^
HTN	165 (72.7%)	148 (72.2%)	17 (77.3%)	0.61
DM	116 (51.1%)	101 (49.3%)	15 (68.2%)	0.14
CKD	10 (4.9%)	9 (4.4%)	1 (9.1%)	0.65
DM & HTN	98 (43.2%)	85 (41.5%)	13 (59.1%)	0.17
DM, HTN, CKD	6 (2.6%)	4 (2.0%)	2 (9.1%)	0.19
CKD Stage				
≤G3a	4 (36.4%)	4 (44.4%)	0 (0.0%)	
≥G3b	6 (54.5%)	5 (55.6%)	1 (50.0%)	
Heart failure	33 (14.5%)	27 (13.2%)	6 (27.3%)	0.07
Previous ACS	17 (28.8%)	16 (29.6%)	1 (20.0%)	0.58
Recent revascularisation (<1y)	15 (24.2%)	15 (26.3%)	0 (0.0%)	
Chronic pulmonary disease	8 (3.5%)	5 (2.4%)	3 (13.6%)	0.83
Peripheral vascular disease	2 (0.9%)	1 (0.5%)	1 (4.5%)	0.05

Most of our patients were admitted with acute coronary syndrome (ACS) (n=209, 92.1%) of which patients who sustained ST-elevation myocardial infarction (STEMI) formed the majority (n=113, 54.1%). Primary PCI (n=208, 91.6%) was the predominant intervention and the majority (n=124, 54.6%) were found to have single vessel disease as demonstrated in Table 3 below. The admitting diagnosis and the findings on intervention were not found to be significantly associated with the development of AKI in our study population. Similarly, the contrast media volume and fluoroscopy time were not associated with the development of AKI.

**Table 3:** Admitting diagnosis and percutaneous coronary intervention-related findings ^1^Median (IQR) or Frequency (%); ^2^Fisher’s exact test, Wilcoxon rank sum test; #Coronary vessels: Number of coronary vessels involved AKI: Acute kidney injury, IQR: Interquartile range, PCI: Percutaneous coronary intervention, ACS: Acute coronary syndromes, CCS: Chronic coronary syndromes, NSTEMI: Non-ST-elevation myocardial infarction, STEMI: ST-elevation myocardial infarction, UA: Unstable angina

Admitting diagnosis & PCI-related findings	Overall n=227^1^	Non-AKI n=205 (90.3%)^1^	AKI n=22 (9.7%)^1^	p-value^2^
ACS	209 (92.1%)	189 (92.2%)	20 (90.9%)	0.83
STEMI	113 (54.1%)	100 (52.9%)	13 (59.10%)	0.36
NSTEMI	58 (27.8%)	52 (27.5%)	6 (27.3%)	0.85
UA	38 (18.2%)	37 (19.6%)	1 (4.5%)	0.11
CCS	18 (7.9%)	11 (5.36%)	2 (9.1%)	0.83
Type of PCI				0.08
Primary	208 (91.6%)	190 (92.7%)	18 (81.8%)	
Non-primary	19 (8.4%)	15 (7.3%)	4 (18.2%)	
#Coronary vessels				0.08
1	124 (54.6%)	115 (56.1%)	9 (40.9%)	
2	62 (27.3%)	56 (27.3%)	6 (27.3%)	
3	41 (18.1%)	34 (16.6%)	7 (31.8%)	
Contrast media volume (in ml)	170 (138,210)	170 (135,210)	200 (140,210)	0.53
Fluoroscopy time (in minutes)	24 (16,35)	24 (16,35)	27 (19,37)	0.49

**Figure 3:**
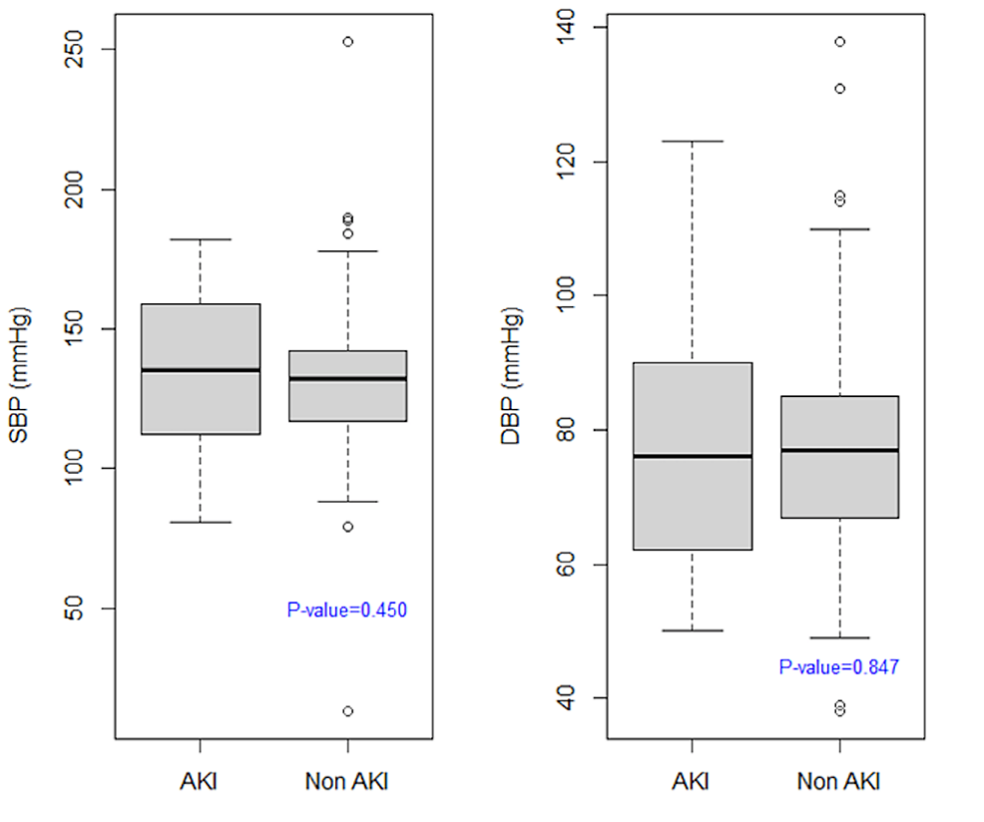
Initial systolic and diastolic blood pressure on admission AKI: Acute kidney injury, DBP: Diastolic blood pressure, SBP: Systolic blood pressure

The median systolic blood pressure (SBP) for individuals who sustained AKI was 136 mmHg (IQR: 112-159) which was slightly higher than those of non-AKI individuals 132 mmHg (IQR: 117-142 mmHg). This was similar to diastolic blood pressure (DBP) measurements where the median for AKI patients was also marginally higher: 76 (IQR: 63-90 mmHg) versus 75 (IQR: 67-85 mmHg). Notably, however, neither of the comparisons showed a statistically significant difference (Figure 3).

Table 4 depicts various laboratory parameters before and after PCI. There was no statistical difference in the AKI versus non-AKI groups when comparing pre-procedural creatinine, hemoglobin, and troponin.

**Table 4:** Laboratory parameters of the study population ^1^Median (IQR) or frequency (%); ^2^Fisher’s exact test, Wilcoxon rank sum test; Δ Creatinine abs: Absolute change in creatinine AKI: Acute kidney injury, IQR: Interquartile range, CKD: Chronic kidney disease, ESRD: End-stage renal disease, PCI: Percutaneous coronary intervention

Laboratory parameters	Overall	Non-AKI	AKI	P-value^2^
	n=227^1^	n=205 (90.3%)^1^	n=22 (9.7%)^1^	
Hemoglobin level, g/dL	14 (13,15)	14 (13,15)	15 (12,15)	0.44
Highest troponin pre-PCI, ng/L	262	248 (33,1738)	1,486	0.16
	(33,1878)		(37,3894)	
Initial serum creatinine, μmol/L	85 (72,101)	84 (72,100)	91 (70,110)	0.38
Baseline creatinine, μmol/L	146	130 (115,165)	252 (252,252)	0.20
(n=10 for CKD/ESRD patients)	(116,191)			
Post-PCI serum creatinine, μmol/L	85 (71,101)	83 (70,95)	129 (116,175)	<0.001
Δ Creatinine abs, μmol/L	0 (-8,7)	-1 (-9,5)	37 (27,87)	<0.001

The majority of the study population (n=185, 81.5%) (p<0.001) had a good outcome. Patients with AKI were more likely to have a poor outcome (59.1%) as compared to those without AKI (14.1%). Amongst individuals who had poor outcomes, a higher percentage with statistical significance was noted in those who had prolonged length of stay >3 days (n=10, 45.5%), amongst those necessitating ICU care (n=5, 22.7%), and in those requiring organ support i.e., hemodialysis (n=5, 22.7%) and ventilator use (n=2, 9.1%). The in-hospital mortality rate in our population was 2.6% and was significantly higher in the AKI group (Table 5).

**Table 5:** Patient outcomes ^1^Median (IQR) or frequency (%); ^2^Fisher’s exact test, Wilcoxon rank sum test AKI: Acute kidney injury, IQR: Interquartile range, LoS: Length of stay

Patient outcomes	Overall n=227^1^	Non-AKI n = 205 (90.3%)^1^	AKI n=22 (9.7%)^1^	p-value^2^
Good outcome	185 (81.5%)	176 (85.9%)	9 (40.9%)	< 0.001
Poor outcome	42 (18.5%)	29 (14.1%)	13 (59.1%)	
LoS >3days	37 (16.3%)	27 (13.2%)	10 (45.5%)	0.0001
Median LoS, days	3 (2,3)	2 (2,3)	4 (3,5)	< 0.001
ICU care	16 (7.0%)	11 (5.4%)	5 (22.7%)	0.003
Ventilator use	5 (2.2%)	3 (1.5%)	2 (9.1%)	0.02
Vasopressor use	8 (3.5%)	6 (2.9%)	2 (9.1%)	0.14
Requiring hemodialysis	5 (2.6%)	0 (0%)	5 (22.7%)	
Death prior to discharge	6 (2.6%)	4.0 (2.0%)	2 (9.1%)	0.047

Table 6 summarises the findings of the multivariable logistic regression analysis. Amongst our study population, none of the clinical, demographic, or procedural characteristics were significantly associated with the development of AKI. 

**Table 6:** Factors associated with acute kidney injury CI: Confidence interval, OR: Odds ratio, LoS: Length of stay, PCI: Percutaneous coronary intervention, DM: Diabetes mellitus

Characteristics	Unadjusted model		Adjusted model	
	OR (95%CI)	p-value	OR (95% CI)	p-value
Sex		0.968	1.60 (0.46-7.54)	0.499
Female	Ref			
Male	1.02 (0.36-3.70)			
Age	1.02 (0.98-1.06)	0.348	1.02 (0.98-1.06)	0.375
DM		0.098	2.15 (0.79-6.45)	0.146
No	Ref			
Yes	2.21 (0.89-5.99)			
Heart failure		0.083	2.62 (0.83-7.56)	0.082
No	Ref			
Yes	2.47 (0.83-6.61)			
Type of PCI		0.092	1.06 (0.81-20.20)	0.956
Non-primary	Ref			
Primary	0.36 (0.11-1.35)			
Troponin pre-PCI		0.809	1.11 (0.27-7.62)	0.898
≤14	Ref			
>14	1.21 (0.32-7.91)			
Vessels involved		0.179	1.35 (0.50-3.68)	0.549
1	Ref			
> 1	1.85 (0.76-4.66)			

## Discussion

This is the first study in Tanzania to document the prevalence of AKI post-PCI. The prevalence amongst our study population was 9.7% (95% CI: 6.2% -14.3%) and 7.9% (95% CI: 4.8%-12.2%) using the CI-AKI KDIGO and AKIN criteria, respectively. Our study displayed a much lower prevalence of AKI post-PCI than a Sudanese study which reported a prevalence of 16.9% [5]. We hypothesize this variance may be due to the difference in race. Our study population was mainly of Asian origin while the population in the aforementioned study was primarily African-dominant. There has been a documented association between African origin and an increased incidence of AKI post-PCI thus necessitating future investigational studies to identify factors and social determinants of this variation [11]. In an Indian study looking at AKI post-PCI, it was found that only 5.6% of study enrollees (amongst the group who had sustained a recent ACS) had worsening renal function post-PCI, and to the contrary, 34.1% had an improving renal function post-PCI [12].

Our prevalence of AKI post-PCI is also comparatively lower than results published globally which range between 16% to 20% [1-3]. The disparity could also be explained by the serum creatinine being affected by BMI, gender, and time to rise. Thus, we advocate the use of novel biomarkers such as cystatin-C, kidney injury molecule-1 (KIM-1), and neutrophil gelatinase-associated lipocalin (NGAL) to provide an accurate reflection of AKI.

On identifying factors associated with AKI post-PCI, none of the variables studied had statistically significant associations. These findings are different from data published globally. Elderly patients are likely to suffer AKI due to decreased renal reserve inhibiting kidney function recovery [13]. Patients suffering from diabetes mellitus have an elevated cardiovascular risk increasing kidney vulnerability to ischemia causing significant microvascular damage. Acute or chronic dysfunction of the myocardium can induce kidney impairment through neuro-hormonal mechanisms and hemodynamic factors [14,15]. Several studies have also reported a higher incidence of all-cause mortality among heart failure patients who sustain AKI post-PCI [16]. The incidence and factors associated with AKI post-PCI among patients suffering from multi-vessel disease are not well understood. Underlying comorbid conditions, the volume of contrast and reduced functioning of the myocardium can be hypothesized as factors. The absence of associations in our study could be due to the small sample size.

Our in-hospital mortality among patients with AKI post-PCI was comparable to data from the NCDR CathPCI®’s large cohort from more than 1000 hospitals in the United States [2]. Our results highlight the need of implementing validated strategies for preventing AKI post-PCI [17]. The majority of our patients who sustained AKI post-PCI had prolonged lengths of hospitalization which is a mere reflection of physiological and hemodynamic compromise of clinical status necessitating organ support and continued care.

## Conclusions

Our study showed that PCI is associated with AKI in approximately 1-in-10 patients in our setting. Notably, locals of Asian ethnicity comprised a majority of the study population: it is reasonable to believe that this may be a factor to explain the relatively lower prevalence of AKI post-PCI in our study compared to other regional and international studies that had a higher proportion study enrollees of Black ethnicity. Nonetheless, whether this was attributable to the demographics of the clientele that the AKH,D caters to; whether this represents the proportion that could afford the costs of treatment (i.e., the PCI procedure); or whether this simply reflects the at-risk population that needs to undergo PCI in the local setting is not clear.

Furthermore, our study has shown that mortality rates for AKI post-PCI are remarkably (x4.5) higher; this is true also with morbidity and complications. Looking to the future, a study with a similar design can be scaled-up to recruit a larger number of participants in a prospective manner and also include more centers both national and regional that perform PCI. Not only will this provide a better understanding and generalisability of post-PCI AKI prevalence, causative factors, associations, and outcomes but may also potentially help identify high-risk individuals and aid in the early initiation of preventive measures.
